# QTL Mapping for Leaf Area of Tea Plants (*Camellia sinensis*) Based on a High-Quality Genetic Map Constructed by Whole Genome Resequencing

**DOI:** 10.3389/fpls.2021.705285

**Published:** 2021-07-29

**Authors:** Yanlin An, Linbo Chen, Lingling Tao, Shengrui Liu, Chaoling Wei

**Affiliations:** ^1^State Key Laboratory of Tea Plant Biology and Utilization, Anhui Agricultural University, Hefei, China; ^2^Yunnan Provincial Key Laboratory of Tea Science, Tea Research Institute, Yunnan Academy of Agricultural Sciences, Menghai, China

**Keywords:** *Camellia sinensis*, leaf area, whole-genome resequencing, genetic map, QTL mapping

## Abstract

High-quality genetic maps play important roles in QTL mapping and molecular marker-assisted breeding. Tea leaves are not only important vegetative organs but are also the organ for harvest with important economic value. However, the key genes and genetic mechanism of regulating leaf area have not been clarified. In this study, we performed whole-genome resequencing on “Jinxuan,” “Yuncha 1” and their 96 F1 hybrid offspring. From the 1.84 Tb of original sequencing data, abundant genetic variation loci were identified, including 28,144,625 SNPs and 2,780,380 indels. By integrating the markers of a previously reported genetic map, a high-density genetic map consisting of 15 linkage groups including 8,956 high-quality SNPs was constructed. The total length of the genetic map is 1,490.81 cM, which shows good collinearity with the genome. A total of 25 representative markers (potential QTLs) related to leaf area were identified, and there were genes differentially expressed in large and small leaf samples near these markers. GWAS analysis further verified the reliability of QTL mapping. Thirty-one pairs of newly developed indel markers located near these potential QTLs showed high polymorphism and had good discrimination between large and small leaf tea plant samples. Our research will provide necessary support and new insights for tea plant genetic breeding, quantitative trait mapping and yield improvement.

## Introduction

The tea plant [*Camellia sinensis* (L.) O. Kuntze] is a perennial, evergreen woody plant originating in southwest China, with a large (approximately 2.84 Gbp) and highly heterozygous genome (Xia et al., 2020a). Many biologically active substances beneficial to the human body, such as catechin, theanine, and caffeine, are abundant in tea, which are important components of tea quality and flavor (Zhao et al., 2018). However, due to the long breeding period and high degree of self-incompatibility, it is difficult to obtain pure line germplasm, explore the formation mechanism of tea quality characteristics, and study the adaptive evolution of different tea populations. In addition, germplasm innovation still faces many difficulties (Meegahakumbura et al., 2016). The construction of a high-density genetic map is of great value for the quantitative trait loci of tea plants, molecular marker-assisted breeding, map-based cloning, and assisted genome assembly.

In the past few decades, based on molecular marker technologies such as simple repeat sequence (SSR) (Hu et al., 2013; Tan et al., 2013; Ma et al., 2014), and amplified fragment length polymorphism (AFLP) (Huang et al., 2005), some tea plant genetic maps have been constructed. However, due to the limitation of the total number of markers and lack of recombinant inbred lines, these genetic maps still have some shortcomings such as poor genome coverage and large gaps, which makes their application in practical research difficult. Fortunately, advances in sequencing technology and reduced costs have made it possible to construct high-density genetic maps. Ma et al. (2015) used specific-locus amplified fragment sequencing technology to obtain 6,448 molecular markers and successfully anchored these markers in 15 linkage groups. Xu et al. (2018) used the F1 population of “Longjing 43” and “Baihaozao” to construct a high-density genetic map with a total length of 1,678.52 cM using RAD sequencing and located 27 QTLs related to catechins or caffeine. However, reduced-representation genome sequencing still has some shortcomings, such as insufficient sequencing depth, uneven distribution of markers, and some pivotal SNP loci related to important traits being undetected. At the same time, different genetic populations will also show different characteristics of segregation. In previous studies, most of the small leaf tea trees were used as parents for hybridization. Due to the insufficient number of harvested F1 generation individuals and the small differences in parental genetic background, the genetic map constructed using these populations is not conducive to accurate QTL mapping for some special traits, such as leaf area, plant type, environmental adaptability, and so on.

The leaves are important nutrient organs and the main harvests of the tea plant, and the delicious tea beverage is processed from the fresh leaves of the tea plant (Tan et al., 2016). The size of tea leaves has an important influence on the yield, especially for black tea (including Pu’er tea), oolong tea, dark tea, and matcha powder. As the two most economically valuable tea types widely planted worldwide, *Camellia sinensis* var. *sinensis* (CSS) and *C. sinensis* var. *assamica* (CSA) have significant differences in leaf area, plant type, cold resistance, secondary metabolic characteristics, and geographical distribution (An et al., 2020). CSA is usually considered to be arborous or semi-arborous with a large leaf area, whereas CSS tea plants are mainly shrubs with small leaf areas. Exploring the key regulatory genes of tea leaf development is of great significance for studying the adaptive evolution of tea trees, breeding new varieties, and increasing the income of tea farmers. In previous studies, Tan et al. (2016) conducted preliminary QTL mapping for the size of mature tea plant leaves, but due to the limited accuracy of the genetic map, it is still necessary to reconstruct a more refined genetic map for QTL mapping based on resequencing.

In this study, the CSS tea plant variety “Jinxuan” was used as the female parent and crossed with the CSA tea plant cultivar “Yuncha 1,” and a total of 96 reliable F1 individuals were harvested. Using whole-genome resequencing technology, two parents and 96 offspring were re-sequenced. Sufficient sequencing data was generated for accurate genotyping. A large number of SNP and indel variations were obtained. Finally, a high-quality genetic map consisting of 15 linkage groups and 8,956 markers was obtained, with a total map length of 1,490.81 cM. Using the F1 generation and the newly constructed genetic map, 25 representative markers (potential QTLs) and two SNPs related to leaf area were successfully located in second linkage group. In addition, we also developed 31 pairs of highly polymorphic indel markers. To summarize, our research not only provides a large amount of basic data and polymorphic loci, but also constructs a high-quality genetic map and QTL mapping for leaf area trait. These data and map will provide strong support for further research on tea plant trait separation, functional gene location and marker-assisted breeding.

## Results

### Identification of SNP and Indel Loci by Resequencing

Whole-genome resequencing produced 1.84 Tbp of raw data. Except for the sequencing depth of the parents, which reached 17×, the average sequencing depth of all the offspring was 5×. Mapping clean reads to the reference genome of “Shuchazao” revealed that the properly_mapped reached 84% (Supplementary Table 1). Sufficient sequencing data and a high alignment rate provide the basis for accurate genotyping.

**FIGURE 1:**
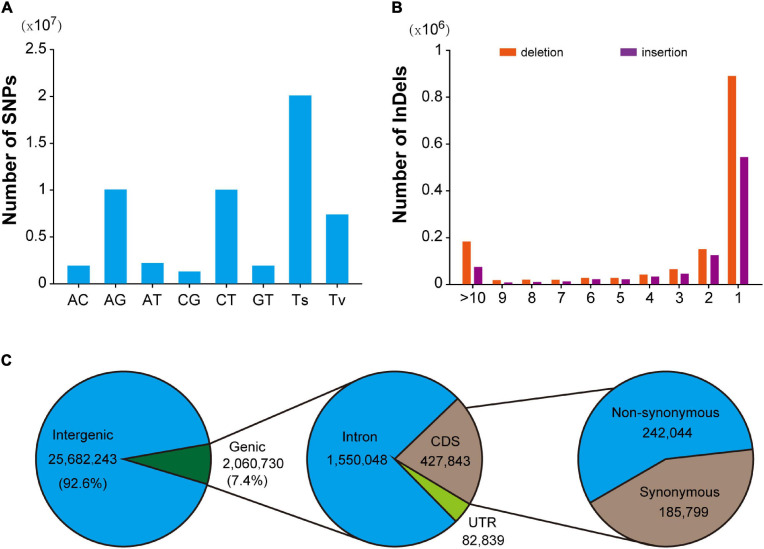
Summary of SNPs and indels detected in the hybrid population. **(A)** Mutation pattern of SNPs in F1 hybrid population. **(B)** Distribution pattern of indel length in F1 hybrid. population. **(C)** Annotation of SNPs identified in hybrid F1 population.

In this population, a total of 28,144,625 SNPs were identified, with an average density of 9.6/Kb. After excluding 648,887 multi-allelic SNPs, it was found that AG and AT mutations were the most abundant types of transformation and transversion mutations, accounting for 35.8% (10,063,296) and 7.9% (2,211,580), respectively. Transformation is the most common mutation type (71.4%), and the ratio of transformation/transversion (ts/tv) is about 2.51 (Figure 1A). In addition, the mutation sites at different positions could have various effects on the function of genes. Detailed location and functional annotations provide more comprehensive SNP variation information. As shown in Figure 1C, of the 27,742,973 annotated SNPs, 92.6% (25,682,243) were located in the intergenic region, whereas only 7.4% (2,060,730) were located in the genes. Of the SNPs located in the genes, 1,550,048, 427,843, and 82,839 were located in the intron, coding region, and untranslated region, respectively. The annotation results showed that 21,373 and 217,884 loci were predicted to have high and medium effects, respectively, and the abnormal gene functions may be caused by these loci. Furthermore, 242,044 SNPs located in the coding region were annotated as non-synonymous mutations. Moreover, 2,780,380 indel mutations were identified using resequencing, including 432,998 multiple allelic mutations. Indels with lengths equal to 1 and greater than 10 accounted for 61.6% and 11% of the total biallelic mutation sites, respectively. Detailed indel length information is shown in Figure 1B. It should be noted that due to the existence of multiple allelic indel loci, it is impossible to annotate all indel mutation sites accurately. However, similar to SNP, most indel variations are also located in intergenic regions.

### SNP Markers Genetic Typing and Genetic Map Construction

**FIGURE 2:**
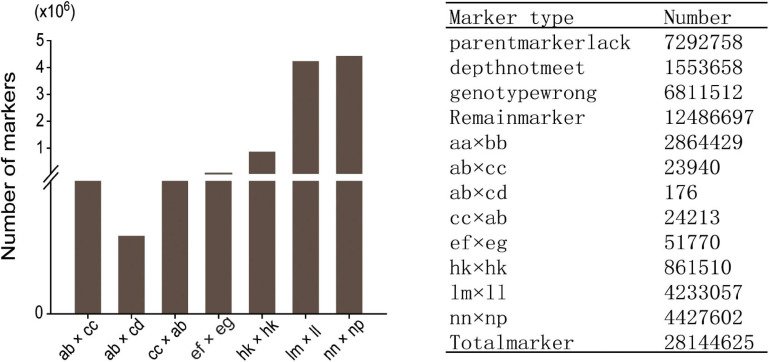
Segregation of SNPs that could be used for genetic map construction.

**FIGURE 3:**
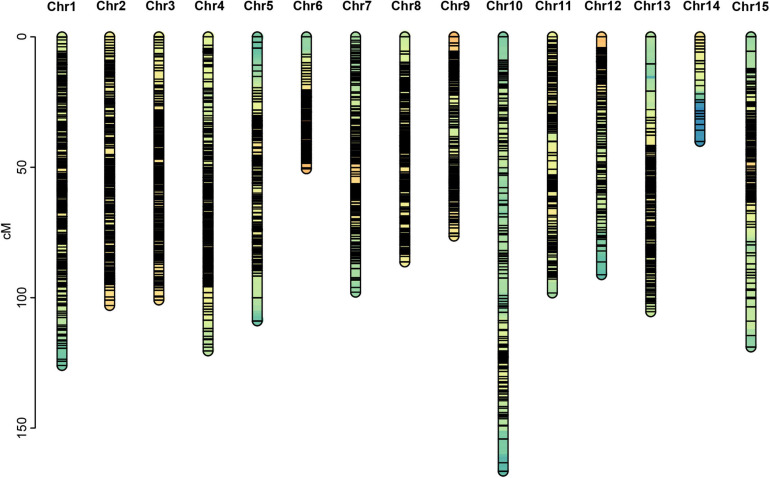
Genetic map constructed in this study.

Among the 28,144,625 markers obtained, after filtering the SNPs of missing parent genotypes (7,292,758), insufficient depths (1,553,658), and genotyping errors (6,811,512), 12,486,697 highly integrated SNPs were obtained. Except for 2,864,429 aa × bb type markers that could not be used to construct a genetic map of the pseudo-testcross population, The remaining 9,622,268 SNPs (including ab × cc, ab × cd, cc × ab, ef × eg, hk × hk, lm × ll, nn × np) were further filtered. As shown in Figure 2, among the 9,622,268 markers, the nn × np type SNP was the most abundant, accounting for 46% (4,427,602), followed by the lm × ll type, accounting for 44% (4,233,057), and the ab × cd type, which was the least abundant, with only 176 SNPs. After strictly filtering the above-mentioned markers and integrating them with a previously reported genetic map, we constructed a high-quality genetic map containing 8,956 markers, with a total length of 1,490.81 cM (Figure 3 and Supplementary Figure 1).

**FIGURE 4:**
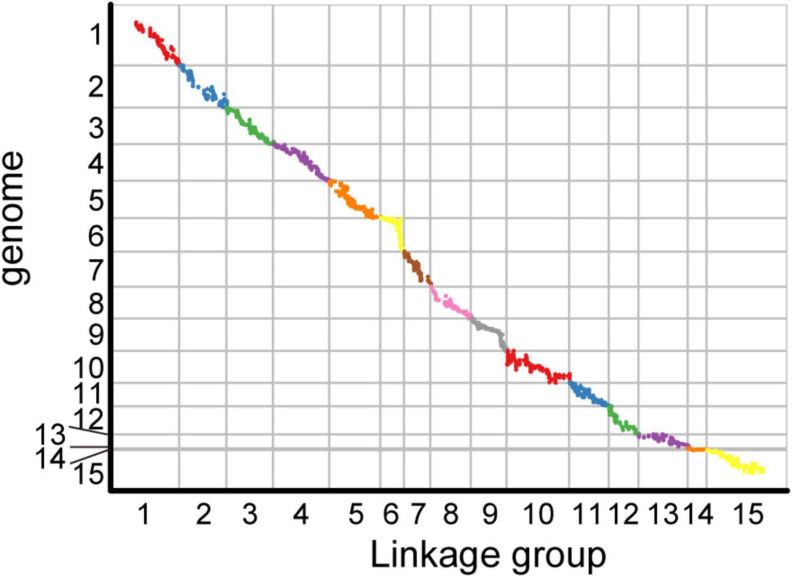
Collinearity analysis of the genetic map and genome based on the arrangement order of markers on genetic map.

Among the 15 linkage groups, the longest one was tenth linkage group, with a length of 166.54 cM, whereas the shortest one was fourteenth linkage group, with a length of only 40.12 cM. Each linkage group contained an average of 597 genetic markers, among which the minimum average map distance and the maximum average map distance appeared in linkage groups 6 and 10, which were 0.1 cM and 0.35 cM, respectively. The maximum gap present in thirteenth linkage group had a length of 10.38 cM, whereas the maximum gap in eight linkage groups was less than 5 cM, indicating that the newly constructed genetic map was high quality. The completeness of the marker data also reflects the quality of the map. In this study, the completeness of all the marker data used to construct the map was over 99%, and there were none missing in the parent genotype. As shown in Table 1 and Figure 4, the closer the spearman coefficient is to 1, the higher the accuracy of the linkage group. Among the 15 linkage groups, the ninth linkage group has the best collinearity with the genome, and the spearman coefficient is as high as 0.993, while the thirteenth linkage group has the lowest collinearity with the spearman coefficient of 0.808. The above results comprehensively indicated that the newly constructed genetic map was high quality and can be used for tea plant genome assembly and QTL mapping research.

**TABLE 1:** Information on the newly constructed genetic map.

**Linkage group**	**Total marker**	**Total distance (cM)**	**Average distance**	**Max gap**	**Spearman**
1	662	126	0.19	4.19	0.992
2	848	103.05	0.12	2.46	0.941
3	929	100.89	0.11	3.22	0.992
4	759	120.45	0.16	3.23	0.989
5	574	108.93	0.19	8.91	0.972
6	506	50.57	0.1	6.68	0.921
7	546	97.93	0.18	3.42	0.944
8	598	86.29	0.14	5.5	0.983
9	612	76.38	0.12	3.31	0.993
10	481	166.54	0.35	9.12	0.91
11	593	98.22	0.17	5.33	0.969
12	482	91.18	0.19	4.87	0.969
13	588	105.39	0.18	10.38	0.808
14	155	40.12	0.26	4.35	0.927
15	623	118.88	0.19	6.68	0.93
Total	8956	1490.81	–	–	–

### QTL Mapping of Leaf Area and Genome-Wide Association Analysis

Leaves are important organs in plants for photosynthesis, respiration, and transpiration. They affect plant growth and development and resistance to adversity in many ways (Ayala et al., 2015). Tea leaves are not only important nutrient organs, but they are also closely related to the yield and quality of tea. According to the leaf area phenotype data of 96 F1 generations (Supplementary Table 2), we used the newly constructed high-density genetic map for QTL mapping. The results are shown in Table 2.

**TABLE 2:** Summary of the information of 25 representative markers (potential QTL).

**Linkage group**	**Position (cM)**	**Representative marker**	**LOD**	**Expl (%)**
2	28.329	Marker1533238	8.11	32.8
2	39.826	Marker1713472	7.89	32.1
2	52.559	Marker1890928	7.89	32.1
2	56.223	Marker1980206	7.78	31.7
2	57.436	Marker2005155	8.02	32.5
2	58.648	Marker2011502	7.68	31.4
2	59.138	Marker1755564	7.89	32
2	59.861	Marker1929075	8.02	32.5
2	61.074	Marker1932620	7.78	31.7
2	68.511	Marker1749183	5.57	23.9
2	70.635	Marker1958387	7.48	30.7
2	70.963	Marker1798200	7.69	31.4
2	72.175	Marker1890445	7.69	31.4
2	74.627	Marker1903871	7.87	32
2	75.839	Marker1943728	7.59	31.1
2	77.052	Marker1999608	8.09	32.7
2	84.744	Marker2104738	6.22	26.3
2	87.186	Marker2092844	6.52	27.3
2	91.08	Marker2050694	7.44	30.5
2	92.045	Marker2197408	7.63	31.2
2	93.925	Marker2201972	7.45	30.6
2	94.798	Marker1892873	7.45	30.6
2	97.223	Marker2048068	6.58	27.5
2	99.674	Marker2048163	5.79	24.7
2	100.887	Marker2057762	6.5	27.3

*LOD, logarithm of odds; Expl, phenotypic contribution rate.*

Twenty-five representative markers related to leaf area were identified from the second linkage group between 28 and 101 cM. Due to the limitation of QTL mapping accuracy, these markers may be regarded as potential independent QTLs. The LOD threshold ranged from 5.57 to 8.11, and the variation contribution rate ranged from 23.9% (potential QTL: Marker1749183) to 32.8% (potential QTL: Marker1533238). All the 25 representative markers were regarded as major loci (variation contribution rate >10%), and the average phenotypic contribution rate was 30.32%. The genome-wide association study (GWAS) is one of the most important methods to study functional genes. In this study, 1,000,682 non-linked disequilibrium high-quality SNPs were used for GWAS analysis, and the results showed that the best model was GLM (Supplementary Figure 2). Interestingly, similar results were observed in two independent analyses, as shown in Supplementary Figure 3, the two leaf area related SNP loci obtained from the GWAS were also located in second linkage group, and the significance test *q* values were 2.9E-8 (L2__134740620) and 5.3E-8 (L2__210876632), respectively. There were 80 genes within the 90 kb interval on both sides of the 25 markers (potential QTL), and most of them have been predicted to be related to cell growth and development and plant organ pathology, including Golgi vessel transport (CSS0032416.2), OVATE family protein 14 (CSS0018166.1), LOC (CSS0013181.1), TSO1-like CXC domain (CSS0001030.1), Phosphatidylinositol-4-phosphate 5-kinase (CSS0011322.1), Sec34-like family protein (CSS0016412.1), ARF guanyl-nucleotide exchange factor (CSS0027335.1), and others. Detailed functional annotations of the 80 genes are listed in Supplementary Table 3.

**FIGURE 5:**
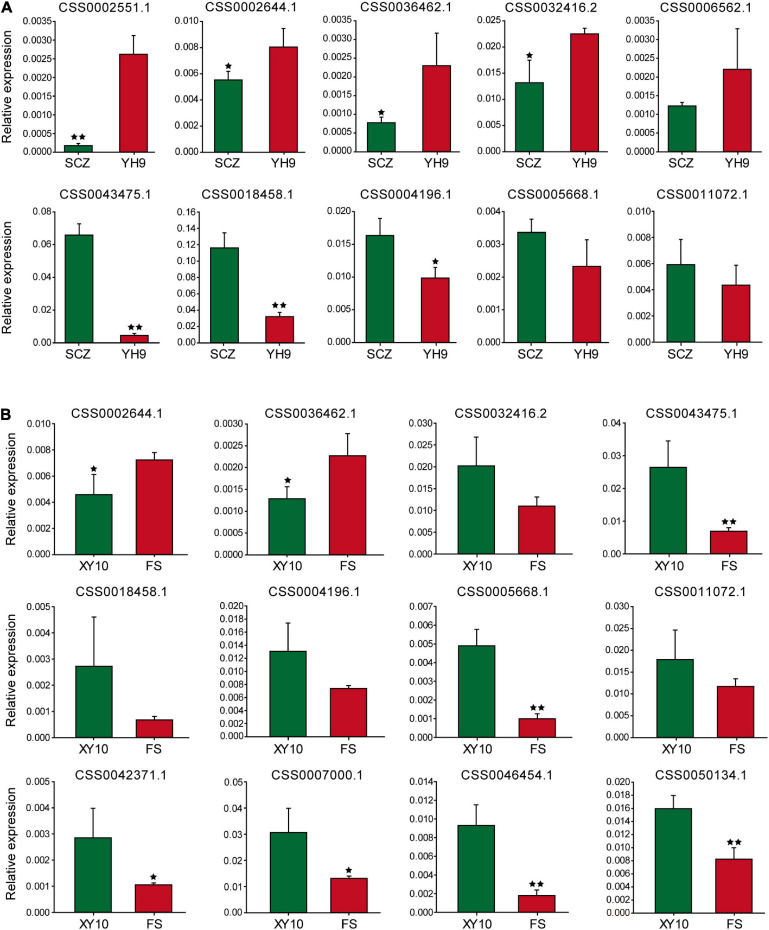
The expression of genes near the potential QTL analyzed using qRT-PCR. **(A)** Shuchazao and Yinghong 9, **(B)** Xinyang 10 and Foshou. * and ** represent significant differences when *p* < 0.05 and *p* < 0.01, respectively.

Sixteen genes were selected for qRT-PCR to explore the expression patterns of these genes in large and small leaf samples. For “Shuchazao” and “Yinghong 9,” as shown in Figure 5A, the expression abundance of 10 genes was successfully detected, and seven of them showed significant differences. Furthermore, two special CSS tea plant varieties, “Xinyang 10” (leaves are narrow and small) and “Foshou” (leaves are round and large) were also used to verify the expression levels of these genes. The expression levels of 12 out of 16 genes were successfully detected (Figure 5B).

Interestingly, the expression patterns of seven genes were similar to their expression characteristics in “Shuchazao” and “Yinghong 9,” suggesting that these genes may indeed be related to leaf development in tea plants.

### Develop Indel Markers Related to Leaf Area

The mutation loci near potential QTLs are thought to be closely related to the growth and development of tea plants. In order to develop genetic markers related to leaf area, we randomly selected 60 indel mutations located within 90 kb of the markers associated with potential QTL to design primers. Based on the initial screening results, after excluding eight primer pairs with invalid amplifications, six primer pairs with non-polymorphic amplifications, and 15 primer pairs with ambiguous amplifications, the remaining 31 primer pairs were further applied in 69 germplasms to verify their transferability and polymorphism and to explore their relationship with leaf area. The amplification results are shown in Supplementary Figure 4. Among the newly developed indel markers (Table 3), InDel17 and InDel18 had low polymorphisms, whereas InDel01, InDel03, InDel09, InDel12, and InDel22 had moderate polymorphisms, and the remaining 24 indel markers were considered to be highly polymorphic (Liu et al., 2019). The genetic diversity of the markers varied from 0.0286 to 0.8354, with an average genetic diversity of 0.622. The PIC and gene diversity were significantly and positively correlated. The observed heterozygosity varied between 0.2903 and 0.7097, with an average of 0.512, whereas the expected heterozygosity ranged from 0.8049 to 0.8604, with an average of 0.837. The marker InDel07 had 12 alleles, whereas InDel17 and InDel18 both had only two alleles. The average number of alleles per marker was 6.48. The MAF varied between 0.2246 and 0.9855, with an average value of 0.5028. Genetic markers with lower major allele frequencies often have higher gene diversity and PIC values.

**TABLE 3:** Sequence and genetic information of the newly developed InDel markers.

**Marker**	**Primer**	**MAF**	**Na**	**Gene diversity**	**Ho**	**He**	**PIC**
InDel01	F:AGCGAGGATTCTGTAGAGGAA R:ATGGTCTCTGCTATCATCCCA	0.6957	5	0.4719	0.4516	0.8482	0.4286
InDel02	F:GCCTTTTCTGCTTTGGGTTTT R:TGTACGATTCGATTCAGTGGG	0.5652	4	0.5878	0.7097	0.8392	0.5245
InDel03	F:GAGTTCAAGATTTGTGTCGTCT R:ATCGTCACCTAGGAATTGGGA	0.7319	5	0.4410	0.5161	0.8287	0.4156
InDel04	F:CACTACTCACAGCCACAAGAG R:TGGATAGGTGGGATCAAGTGA	0.3768	11	0.7863	0.4516	0.8408	0.7623
InDel05	F:GATCCGGTGCTATCATTACCC R:AACCCTGAACAACTCTTTCCC	0.3768	6	0.7418	0.6774	0.8361	0.6998
InDel06	F:ATGCATGCCCTTGAACTAGTT R:GACAAGTCACAAGCAGCAAAG	0.3551	10	0.7770	0.5484	0.8604	0.7482
InDel07	F:ACCAATGATTGTTCTGCACCT R:ACAAACCCCACTACAAAAGCT	0.5000	12	0.6839	0.4516	0.8186	0.6497
InDel08	F:CCAGTGATCGATCGAATTGGA R:GCATGAAAAAGCGCGCAATAA	0.4783	9	0.6596	0.6129	0.8456	0.6057
InDel09	F:TGAGAAGGACCAGAGTGAGAA R:TGACTGCCCAAATTTAGAGCT	0.6739	4	0.4933	0.5161	0.8371	0.4443
InDel10	F:CACATTGGTAAGATAGCCCTT R:AGGCCTCTTTGTTTAGTTGCA	0.4058	5	0.6577	0.5806	0.8318	0.5932
InDel11	F:CTAAGGCCACAAACATGCTTG R:ACAATATTGGCAGCTCAGGTT	0.5145	4	0.6142	0.5806	0.8281	0.5442
InDel12	F:GTACGCTCAATTTGGTGTTCG R:CAATTCAAGTCAAAGCCAGGC	0.6014	6	0.5564	0.5161	0.8519	0.4962
InDel13	F:AATGGGCAAAAACAATGGAGC R:CCAAAACAAAACCCAAAGCAG	0.4420	6	0.6971	0.6452	0.8525	0.6503
InDel14	F:TTTGGCCACTCGAAAATGTTC R:GAGGAGTTCTTTGTGTGGGTT	0.4565	4	0.6612	0.5161	0.8498	0.6015
InDel15	F:GAAGGCAAAGCAAGATGATGG R:ATGCAGCTTCTACTTCCCAAG	0.3043	9	0.7990	0.5161	0.817	0.7712
InDel16	F:GTAAAGTATGCAGGCTCCGAA R:TTGGACCCAGCTACTTCAATG	0.4783	5	0.6796	0.5806	0.8361	0.6329
InDel17	F:GTTTGGCGTTAGATGTGCATT R:ACTTGGATCAGCTTGGAATGG	0.9855	2	0.0286	0.5161	0.8049	0.0282
InDel18	F:ACATTGGTCATTTCCCCTTCA R:AGGCTTTGATGGTTTGGTCAT	0.9783	2	0.0425	0.4194	0.8186	0.0416
InDel19	F:CAACAGCAACACCACTAACAC R:GCACTTCACACCTCAACAATG	0.5652	8	0.6133	0.4516	0.8149	0.5681
InDel20	F:ACTAGTTGCTTTGCTTCGCTA R:TAAGCATCATGAAACACGGGT	0.3623	4	0.6706	0.4194	0.8546	0.6003
InDel21	F:AATATTCGGCAGTGTGTTTCC R:TCAGGTCCGAACAATAGAAACT	0.4058	10	0.7438	0.5806	0.8361	0.7093
InDel22	F:CTGATGCTTCCGACCATATGT R:AGAGGAATGAGCCAACTTGTC	0.4783	3	0.5843	0.3871	0.8451	0.4952
InDel23	F:ATTTGGTTGCAGGAAGAGGTG R:GTGGTGATGGGGATTTGGTAA	0.5870	6	0.5735	0.5161	0.8408	0.5161
InDel24	F:TGCAATTTAGGGGCCACATTA R:GTCCCCTTTCATGAACCACTC	0.5797	7	0.6067	0.4516	0.8197	0.5672
InDel25	F:CACCACAAACAAACACCCAAA R:AGGTCTCCTCCATCCAAAGAA	0.2246	9	0.8354	0.4839	0.8228	0.8145
InDel26	F: CGACCTATCCCTTTTCCCA R: GGTTCCACGCAGATTTCAC	0.4275	8	0.7049	0.4194	0.8176	0.6579
InDel27	F:TTATGGCGAGTTTCGATCTG R:TCTGTAGGAGTCTCTGTGGT	0.5942	4	0.5752	0.4839	0.8435	0.5222
InDel28	F:ACCGTTGGAAATGCTCTTAGG R:CATGCGCATAAACGGCTTAAT	0.3188	10	0.8240	0.5484	0.8451	0.8046
InDel29	F:TCCTTTCGATCTGGCATGTTT R:TAGCCAGCCTATAGAACGACA	0.2391	11	0.8043	0.4839	0.8599	0.7758
InDel30	F:TTCCATAGTATTGCCGCCTTG R:GGATCTTTGTGCCTCTCACCG	0.4928	5	0.6332	0.2903	0.8599	0.5666
InDel31	F:AATCTCTCCGCCAGCAATACC R:GGACGAAAACCAAATAACTCA	0.3913	7	0.7476	0.5484	0.853	0.7109
Mean	–	0.5028	6.48	0.6224	0.512	0.837	0.5789

*Na, number of alleles; MAF, major allele frequency; Ho, observed heterozygosity; He, expected heterozygosity; PIC, polymorphism information content.*

### Phylogenetic and Population Structure Analysis

**FIGURE 6:**
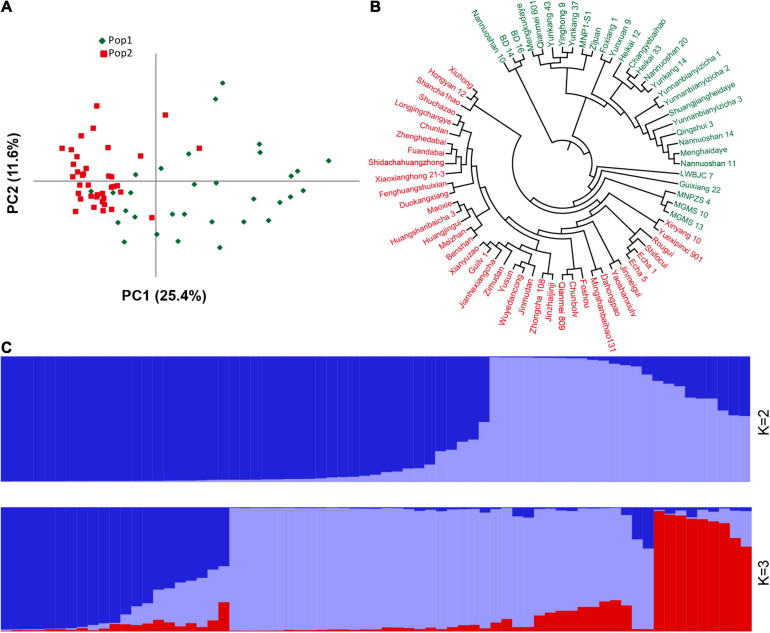
Population structure and phylogenetic analyses of 69 germplasm samples based on the newly developed indel markers. **(A)** PCA analysis. **(B)** UPGMA phylogenetic tree. **(C)** Population stratification. For **(A-B)**, green represents Pop1, red represents Pop2, and for **(C)**, different colors represent different population stratification.

There are several of SNP and indel mutations in the vicinity of the potential QTL, and some genomic mutations located in regulatory or coding regions may be related to the development of plant leaves. Therefore, based on the newly developed polymorphic indel markers, we performed population structure and principal component analyses on 69 tea resources. The results showed that the best model for the 69 tea tree samples was achieved when K was set to 2 (Supplementary Figure 5). Most of the samples were correctly classified together based on different germplasm types when K was 2. As shown in Figure 6, the first category contained the most CSA samples, whereas the second category was mainly composed of CSS samples. In particular, when K was set to 3, wild germplasm samples from different regions were clearly classified, implying that our newly developed markers have good discrimination capabilities. Similar clustering results were observed using a PCA (Figure 6A). The variation explanation rates of the first and second principal components were 25.4 and 11.6%, respectively. Furthermore, based on the genetic distance of the newly developed genetic markers, we constructed a phylogenetic tree composed of 69 tea tree samples with the UPGMA model. All the samples were classified into two groups based on a phylogenetic analysis (Figure 6B). Similar to the results of the population structure and principal component analyses, tea varieties with larger leaf areas were assigned to the first group, whereas tea varieties with smaller leaf areas were assigned to the second group (Figures 6A,C). The accurate classification results suggest that the newly developed genetic markers and the genes in which these markers are located may be related to the development of tea leaves.

## Discussion

Genetic maps are widely used for QTL mapping of key genes of many important agronomic traits (Toojinda et al., 2003; Liu et al., 2015; Ren et al., 2018). Most of the early genetic maps were constructed using randomly amplified polymorphic DNA (RAPD) (Ota, 1999), simple sequence repeats (SSR) (Yamamoto et al., 2005), inter-simple sequence repeats (ISSR) (Kojima et al., 1998), AFLP (amplified fragment length polymorphism) (Marsan et al., 2001), and other molecular markers (Jarvis et al., 1994). Low marker density and large genetic distances make it difficult to apply these genetic maps in practical research. In recent years, with the advancements in technology and the reduction in sequencing costs, large-scale resequencing has been made possible. A large number of genetic markers can be mined using extensive resequencing, which provides strong data-based support for the construction of high-density genetic maps. It was not until 2018 that the genome of the first small-leaf tea plant “Shuchazao” was released (Wei et al., 2018), which greatly promoted basic research on tea trees. However, the underlying mechanisms of many important agronomic traits in tea trees have not yet been revealed (Xia et al., 2020b). The construction of a high-density genetic map is still of great significance for anchoring these markers in tea plant functional genes and breeding of excellent varieties. In recent years, some high-density genetic maps of tea plants have been reported. For example, Xu et al. (2018) constructed a genetic map using 2b-RAD sequencing with the hybrid population of Longjing 43 (“LJ43”) and Baihaozao (“BHZ”); based on specific-locus amplified fragment sequencing, Ma et al. (2015) constructed a genetic map with Yingshuang (“YS”) and Beiyue Danzhu (“BD”) as parents. Although these genetic maps have high marker density because most of the mapping parents belong to CSS varieties, there are still some restrictions on QTL mapping and map-based cloning.

In this study, the whole genome of the CSS tea variety “Jinxuan,” CSA tea variety “Yuncha 1” and their 96 hybrid F1 generations were re-sequenced, and a total of 1.84 Tb of raw sequencing data were obtained. The sequencing depths of the parents and offspring reached 17× and 5×, respectively. Of the clean reads, 84% could be well-aligned to the genome. Compared with previous studies (Su et al., 2017; Rehman et al., 2020), genome-wide resequencing can provide more abundant raw data than ever before. All clean data were submitted to GATK-HaplotypeCaller for mutation detection. In this population, 30,925,005 variations, including 28,144,625 SNPs and 2,780,380 indels, were identified. The average SNP density was 9.6/Kb, which is much higher than that previously reported (An et al., 2020). Similar to other crops (Shen et al., 2017; Thakur and Randhawa, 2018; Muñoz-Espinoza et al., 2020), transitions had an absolute advantage in SNPs, and the ts/tv value was approximately 2.51, which was higher than Cucurbita (Xanthopoulou et al., 2019) but lower than lotus (Huang et al., 2018). Moreover, 242,044 and 185,799 SNPs were annotated as non-synonymous mutations and synonymous mutations, respectively. The ratio of the non-synonymous_variant to the synonymous_variant was approximately 1.3, which was higher than those for watermelons (Guo et al., 2019) and sweet potatoes (Zhang et al., 2020), but equivalent to that for rice (Yano et al., 2016).

After a series of filtering of 28,144,625 SNPs, 12,486,697 highly integrated SNPs were obtained. Since 2,864,429 aa × bb type markers could not be used for the construction of the pseudo-testcross population genetic map, the remaining 9,622,268 SNPs were used for further genetic analysis and genotyping. Among the 9,622,268 SNPs, the most abundant types were nn × np and lm × ll, accounting for 46 and 44% of the total, respectively. Finally, a high-quality genetic map containing 8,956 markers with a total length of 1,490.81 cM was successfully constructed. These markers were relatively and evenly distributed across 15 linkage groups, and each linkage group contained an average of 597 genetic markers. The genetic map evaluation revealed that eight of the 15 linkage groups had a maximum gap of less than 5 cM. The integrity of all the markers used in the genetic map exceeded 99% and the parental genotype was complete. A collinearity analysis of the genetic map and genome is also commonly used to evaluate the quality of each linkage group. It was found that the newly constructed genetic map and genome had superior collinearity, with an average spearman coefficient of 0.95. These results indicated that the genetic map constructed in this study was high quality and could be applied to QTL mapping and marker-assisted breeding in tea plants. Compared with the other previously reported genetic maps of tea plants, the total map length here was moderate and contained most of the genetic markers (Ma et al., 2014, 2015; Xu et al., 2018). To the best of our knowledge, this the first high-quality genetic map based on the hybrid population of CSS and CSA constructed using whole-genome resequencing technology.

Leaves are important vegetative organs in plants and provide energy for the growth and development of plants through photosynthesis. Fresh tea leaves have important economic value because they can be made into a popular beverage with unique flavor (Tai et al., 2015; Xu et al., 2017). CSS and CSA are the two most widely distributed and used tea plant varieties (Wang et al., 2020). However, due to the impacts of many factors such as geographical distribution, environmental adaptation, and artificial selection, they are different in many aspects such as morphological characteristics and content. Most notably, CSA variants tend to have a larger leaf area compared to CSS, and this difference has not been clearly explained. We identified 25 representative markers and two SNPs related to leaf area via QTL mapping and GWAS analysis, which distributed in the second linkage group between 28 and 101 cM. Notably, 25 representative markers may indicate some potential QTLs that have not been identified considering of the small scale of mapping population and elongated QTL shape. All the 25 representative markers (potential QTL) were regarded as major loci (variation contribution rate >10%) with LOD values ranging from 5.57 to 8.11. A significant SNP (L2__210876632, *q* value 5.3E-8) obtained using GWAS was only 1.3 Mb away from potential QTL Marker2197408. Similar results that were obtained using different methods suggested that the QTL mapping was reliable. Many genes that may be related to tea leaf development were identified near the potential QTL. For example, Wang et al. (2018) showed that the CPP-like gene family in Arabidopsis (CSS0001030.1) can expand cells and differentiate buds, and Zhou et al. (2021) found that OVATE family protein genes (CSS0018166.1) can regulate peach fruit traits and leaf shape. In order to further explore the expression patterns of these genes in large-leaf and small-leaf tea plants, 16 genes were selected to verify their expression levels using qRT-PCR. The results showed that there were some genes that were significantly and differentially expressed in the large leaf varieties and small-leaf varieties, and the expression trend was related to leaf area and not to whether it was CSS or CSA. Interestingly, the expression level of these genes was very low, which may indicate that the development of tea leaves is regulated more by minor genes.

There are many genetic mutations in the plant genome that play an important role in plant growth and development. Zhou et al. (2021) identified a 1.7 Mb chromosomal inversion that is responsible for regulating the shape of peach fruit; Cheng et al. (2019) found that a single nucleotide mutation in GID1c can dwarf peach trees; Wang et al. (2016) found that genetic variations in ZmVPP1 contributes to drought tolerance in maize seedlings by GWAS. It is these mutations that enhance genetic diversity within species. In this study, based on sufficient genetic variations provided by extensive resequencing, we developed 31 pairs of indel markers related to leaf area located near QTL. Most of these markers located in genes, which may alter gene function. The average gene diversity and PIC were 0.6224 and 0.5789, respectively. Twenty-four of the 31 markers had high polymorphism and good mobility. Based on these markers, we analyzed the population structure and heredity of various tea germplasm samples. The results showed that 69 tea plant resources with different genetic backgrounds were mainly classified into two groups. When *K* = 3, obvious genetic structure was observed, and wild tea plant germplasm samples and cultivated tea plants had significantly different lineages. In the phylogenetic tree, 29 CSA varieties and one CSS species “Guixiang 22” were divided into the first category, whereas 37 CSS varieties and two CSA varieties “Xiuhong” and “Qianmei 809” were divided into the second group. Considering that one parent of “Guixiang 22” belongs to the C. sinensis var. pubilimba variety and that “Xiuhong” and “Qianmei 809” each have a parent belonging to the CSS class of tea plants, this classification may be reasonable. This result is also supported by the population analysis results.

The growth and development of plant leaves are controlled by many factors, such as light, temperature, disease, nutritional status, and transcriptional regulatory genes (Franklin, 2009; Vega et al., 2019; García-Gómez et al., 2020; Li et al., 2020). For example, Sun et al. (2020) found that OVATE family protein 6 can regulate the angle of rice leaves and Michael (Muszynski et al., 2020) found that cytokinin can change the development pattern of maize leaves. Long-term hybridization means that most cultivated tea trees have complex genetic backgrounds, which brings many challenges for studying the genetic domestication of tea trees and locating key genes that control important agronomic traits. One of them is that the developmental mechanism of tea leaves has not been clearly revealed. In our study, the 31 pairs of polymorphic markers that were developed based on QTL mapping results could divide most tea plants according to their leaf area. However, some special tea varieties, such as “Foshou” had even larger leaf areas than some CSA varieties, but they were clustered together in the second main branch of the phylogenetic tree. Since quantitative traits are often regulated by multiple genes, this result suggested that there might be more unidentified genetic regions that could regulate the development of tea leaves.

## Conclusion

In this study, we performed whole-genome resequencing on “Jinxuan,” “Yuncha 1,” and their 96 hybrid F1 samples and identified a large number of SNPs and indel mutation loci. A high-quality and high-density genetic map of tea plants with good collinearity with the reference genome was successfully constructed. Using QTL mapping and GWAS, 25 representative markers (potential QTL) were identified, which distributed in the second linkage group with an average phenotypic contribution rate more than 30% and containing two SNP mutations related to leaf area. A total of 31 polymorphic indel primers near the potential QTL were developed, and PCR was performed in 69 diverse tea germplasm samples to detect the mobility and discrimination capabilities of these markers. Population structure and phylogenetic analyses showed that the newly developed markers can better distinguish large-leaf species from small-leaf species. However, due to their complex genetic background, there may be more unknown genetic regions in the genome that may play an important role in regulating the development of tea leaves. To conclude, in this study, besides preliminary QTL mapping of genes that regulate tea leaves, the newly constructed genetic map and developed molecular markers will provide basic support for tea breeding and quantitative trait research.

## Materials and Methods

### Plant Materials

Ninety-six hybrid F1 generations and their parents “Jinxuan” and “Yuncha 1” were cultivated in the national germplasm repository of large-leaf tea in Menghai, China. The young buds and leaves were picked and quickly frozen and subsequently stored in a −80°C freezer until they were sequenced. Another 69 germplasms from different countries and regions with different leaf shapes were used to evaluate the newly developed genetic markers. Detailed information on these samples was listed in Supplementary Table 4.

### DNA Extraction and Whole-Genome Resequencing

The DNA of all the samples was extracted from young leaves using a modified CTAB method, and the extracts were electrophoresed on a 0.8% agarose gel to check their quality and integrity. Qualified DNA samples were broken into short fragments of 200–500 bp using ultrasound, and then paired-end sequencing was performed on F1 generations using the Illumina Hiseq 2500 sequencing platform. Using the Trimmomatic software, the original data were filtered according to the following standards: (1) remove adapters, (2) remove paired reads if the ratio of N in the sequenced fragments is greater than 10%, and (3) remove reads if more than 50% of the bases had a base quality value *q* < 10 in the sequenced fragments and ensure that the bases with *Q* > 20 are above 90%.

### Detection and Filtering of Genetic Variations and Construction of Genetic Maps

The filtered reads were aligned to the reference genome “Shuchazao” through the built-in MEM module of the BWA (Burrows-Wheeler Aligner) program. The Picard script was used to remove PCR duplications in the BAM files (Liu et al., 2019). These files were then submitted to GATK-HaplotypeCaller for mutation detection (Niu et al., 2019). The raw mutation site files of each sample were merged and filtered according to the following criteria: (1) two SNP sites within 5 bp, (2) SNP sites within 10 bp near indel, (3) two indel with a distance less than 10 bp, (4) for diploid species, one SNP marker locus can contain at most four genotypes, so SNP loci with more than four alleles were discarded subsequently. Only SNPs with two to four alleles were identified as polymorphic and considered potential markers. All polymorphism SNPs loci were genotyped with consistency in the parental and offspring SNP loci. In order to facilitate analysis, all the SNP locus genotypes were coded following the two-allele coding rules commonly used in genetics. Among them, the aa × bb genotype locus was directly removed because it was not suitable for constructing a genetic map in this project. The remaining SNPs were filtered according to the following criteria to ensure the quality of the map: (1) the sequencing depth of the parents is less than 10×, (2) the sequencing depth of the offspring is less than 2×, (3) the completeness of the genotype is less than 90%, (4) the *p*-values of segregation distortion are less than 0.05, and (4) the SNPs have MLOD values lower than 3. With the help of the HighMap software (Xu et al., 2015), the linear arrangement of the markers in the linkage group was obtained, and the genetic distance between the adjacent markers was estimated. At the same time, the previous map (Xu et al., 2018) were integrated using the BioMercator (Sosnowski et al., 2012) software, and finally, the high-quality genetic map was drawn. Besides, vcftools was used to perform statistical analysis on the final variations. Based on the index file of the “Shuchazao” genome constructed using SnpEff, all the variation sites were annotated and classified.

### QTL Mapping and GWAS

The length and width of the six mature leaves of each F1 individual were accurately measured, and the leaf area of each leaf was calculated according to the formula: leaf length × leaf width × 0.7 (NY/T 1312-2007), and then the average of the six leaf areas was taken as the leaf area of the offspring. The built-in interval mapping method of the MapQTL software was used to locate the QTL of the leaf area, and all the default parameters of the software were used. With the PLINK software, all the SNP loci were filtered under the conditions of -maf 0.05, -geno 0.1, and -indep-pairwise 50 10 0.2. Based on the 1,000,682 high-quality SNP loci of non-linkage disequilibrium obtained after filtering, the three models (including GLM, MLM, and CMLM) provided by the TASSEL (Bradbury et al., 2007) software was used for the GWAS. Similarly, the kinship and principal component analysis (PCA) files of all the samples were calculated under the default parameters of the TASSEL software. The Manhattan map and linkage group were drawn using the CMplot and LinkageMapView packages in R.

### RNA Extraction and Quantitative Real-Time PCR (qRT-PCR)

The young second leaves of “Shuchazao” and “Yinghong 9” were collected from the Tea Tree Resource Nursery of the Anhui Agricultural University, and their total RNAs were extracted using the Total RNA Purification Kit (Norgen Biotek Corporation, Canada) following the protocol of the manufacturer. In addition, a CSS variety called “Foshou” which has a very large-leaf area, and a CSS variety called “Xinyang 10” which has a small leaf area, were also collected from the Guangdong province and their RNAs were extracted as described above. Subsequently, first-strand cDNA was synthesized based on the qualified RNA using a PrimeScript RT Reagent Kit (cat 6110A, Takara, Japan) for qRT-PCR. Detailed PCR amplification and thermal cycling conditions were performed referring to our previous research. The relevant primer sequences are listed in Supplementary Table 5.

### Development of Indel Markers

In the 2 Mb region before and after potential QTL, indels with a length ≥4 were used as candidate sites. The 300 bp double-flanking sequence of the indel was extracted as the primer design region. The primers were designed using Primer3 based on the following criteria: amplicon lengths should range from 150 to 350 bp; primer lengths, 20–22 bp, with the optimum length being 21 bp; Tm 55–60°C, with 58°C being the optimum, and the maximum difference between the annealing temperatures of the primer pairs should not exceed 5°C; GC content 40–60%, with 55% being the optimum. A total of 60 indels located near the potential QTL that met the above conditions were randomly selected, and the primers were synthesized. Eight germplasms, including “Hekai10,” “Hekai38,” “Nannuoshan 9,” “Nannuoshan 13,” “Yaoshanxiulv,” “Shidacha 1,” “Xiaoxianghong 21-3,” and “Echa 1” were used to screen all the primers preliminarily using the Fragment Analyzer^TM^ 96 system (Advanced Analytical Technologies, Inc., Ames, IA, United States). More detailed amplification conditions and primer screening can be found in our previous study.

### Genetic Diversity, Population Structure and Phylogenetic Analysis

The selected primers were further applied to 69 diverse tea plant samples to verify their mobility and polymorphism. The amplification results were displayed graphically using prosize2.0 built in the Fragment Analyzer^TM^ 96 system, and the amplified fragment length was identified and converted into letter codes. Subsequently, the expected heterozygosity (He), observed heterozygosity (Ho), number of alleles (Na), major allele frequency (MAF), and polymorphism information content (PIC) were also calculated using PowerMarker3.25 and PopGene32. Concurrently, the phylogenetic tree of 69 diverse tea plant samples was also constructed using the unweighted pair-group method with the arithmetic means (UPGMA) algorithm using PowerMarker at the default setting of 1,000 bootstraps. The population structure analysis was performed with reference to previous reports (Liu et al., 2019). A web-based online software, Structure Harvester (http://taylor0.biology.ucla.edu/structureHarvester/), was used to evaluate the best population *k* value. GenAlEx 6.5 (Liu et al., 2017) was used to perform the PCA analysis.

## Data Availability Statement

The original contributions presented in the study are publicly available. This data can be found here: NCBI database, BioProject ID is PRJNA727668.

## Author Contributions

YA performed the data analysis and manuscript drafting. CW and SL conceived the project and designed the research. LC and LT performed the hybridization, experiments, interpreted the results, and analyzed the data. All authors have read and approved the manuscript.

## Conflict of Interest

The authors declare that the research was conducted in the absence of any commercial or financial relationships that could be construed as a potential conflict of interest.

## Publisher’s Note

All claims expressed in this article are solely those of the authors and do not necessarily represent those of their affiliated organizations, or those of the publisher, the editors and the reviewers. Any product that may be evaluated in this article, or claim that may be made by its manufacturer, is not guaranteed or endorsed by the publisher.

## Supplementary Material

Supplementary Figure 1 Location information of markers in the genetic map.

Supplementary Figure 2 Manhattan and QQ plots of GLM, MLM, and CMLM models.

Supplementary Figure 3 The position of SNP loci on the second linkage group obtained by GLM model.

Supplementary Figure 4 Amplification polymorphism of 31 pairs of indel markers among the 69 germplasm samples.

Supplementary Figure 5 Estimation of the optimal group number through ΔK. The value of K was set from 2 to 8.

Supplementary Table 1 Summary of the sequencing data of the F1 generation and their parents.

Supplementary Table 2 Average leaf area information for each offspring.

Supplementary Table 3 Annotation information of genes within 90 kb of both sides of the 25 representative markers (potential QTL).

Supplementary Table 4 Tea plant samples information used in indel marker development.

Supplementary Table 5 Primer information used for qRT-PCR.

## Abbreviations

QTL: Quantitative trait locus
SNP: Single nucleotide polymorphism
qRT-PCR: Quantitative real-time PCR
indel: Insertion-deletion
LOD: Logarithm of odds.
