# Supplementary figures and images for: QTL Mapping for Leaf Area of Tea Plants (Camellia sinensis) Based on a High-Quality Genetic Map Constructed by Whole Genome Resequencing
Source: Front Plant Sci. 2021 Jul 29;12:705285. doi: 10.3389/fpls.2021.705285 (PMC8358608; doi:10.3389/fpls.2021.705285)

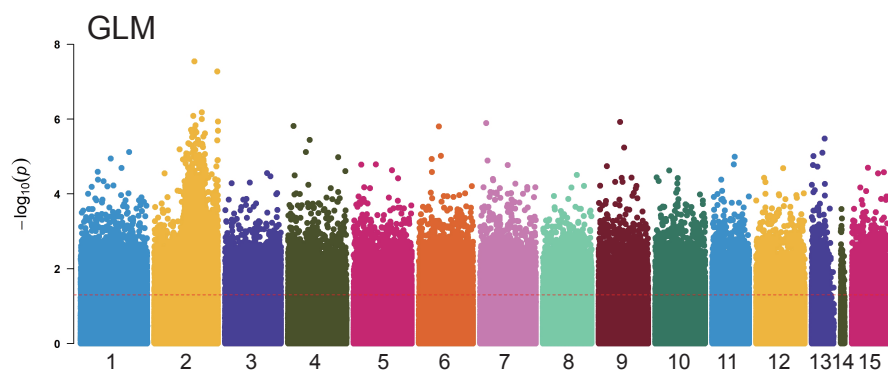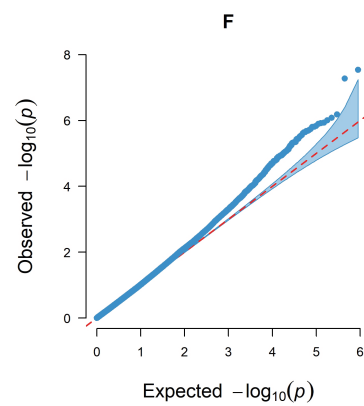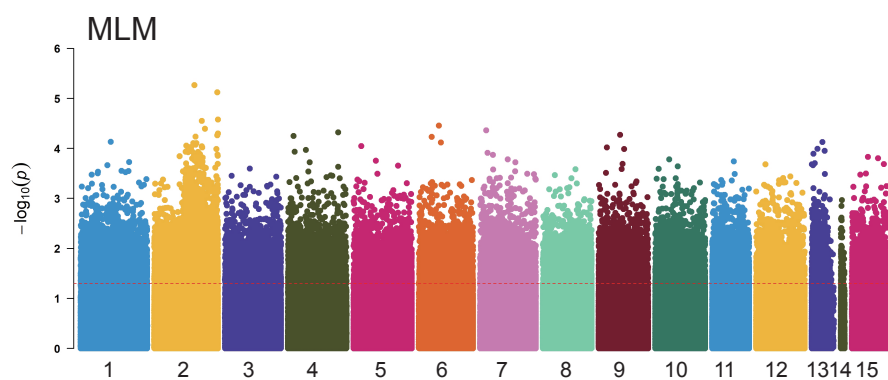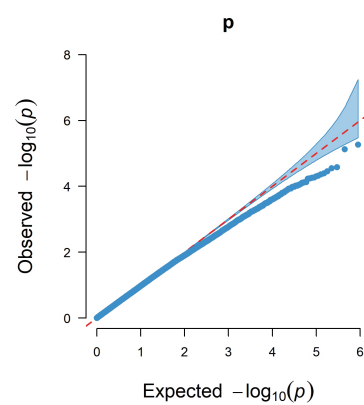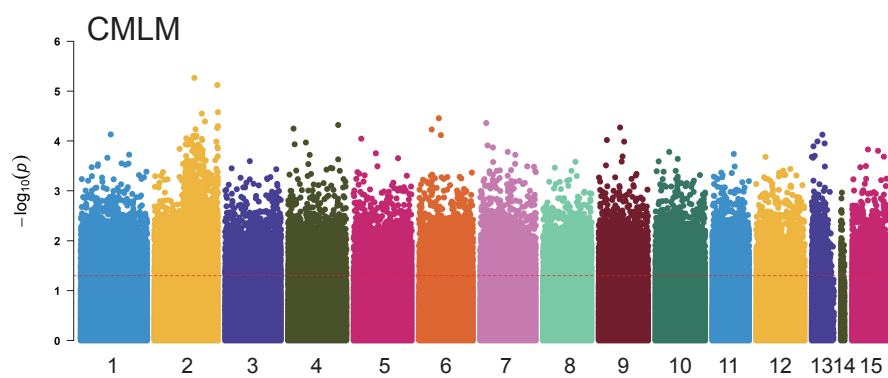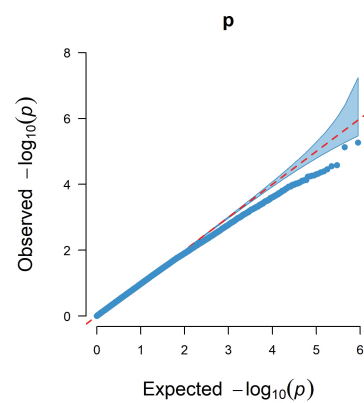

Supplement: Supplementary Figure 2 — Manhattan and QQ plots of GLM, MLM, and CMLM models. [file Data_Sheet_2.PDF]

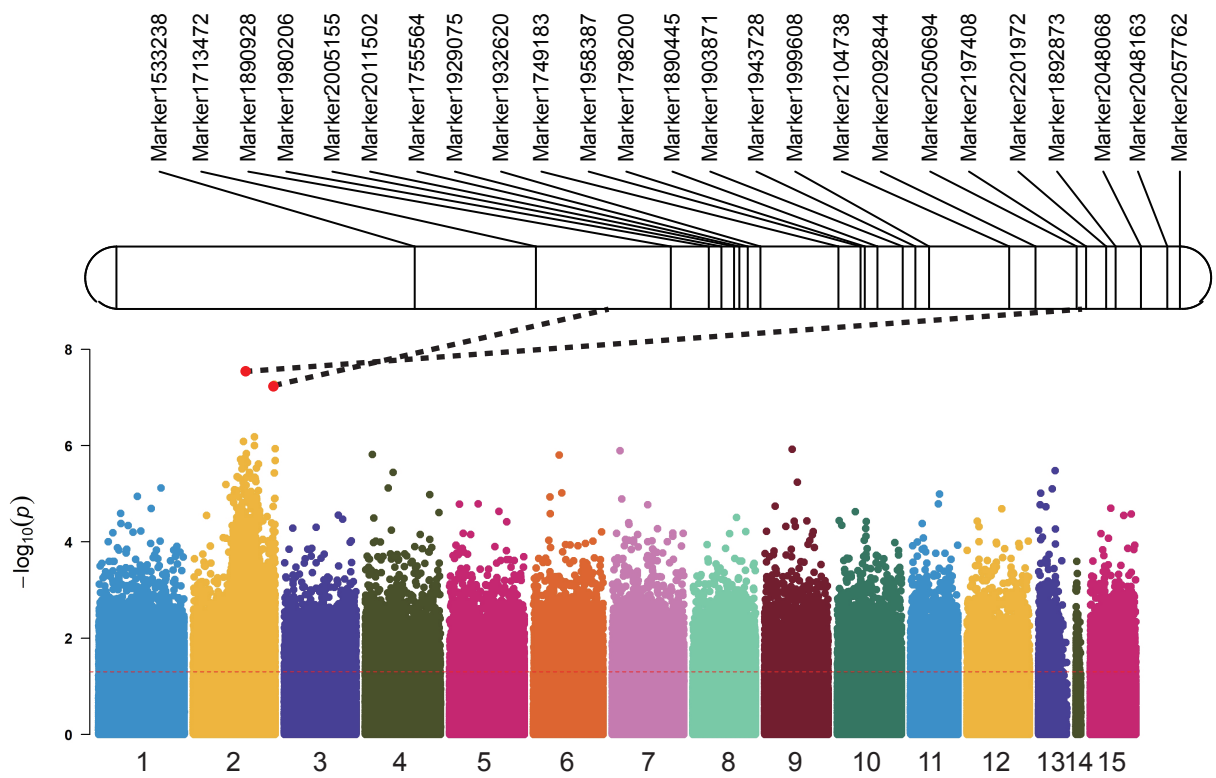

Supplement: Supplementary Figure 3 — The position of SNP loci on the second linkage group obtained by GLM model. [file Data_Sheet_3.PDF]

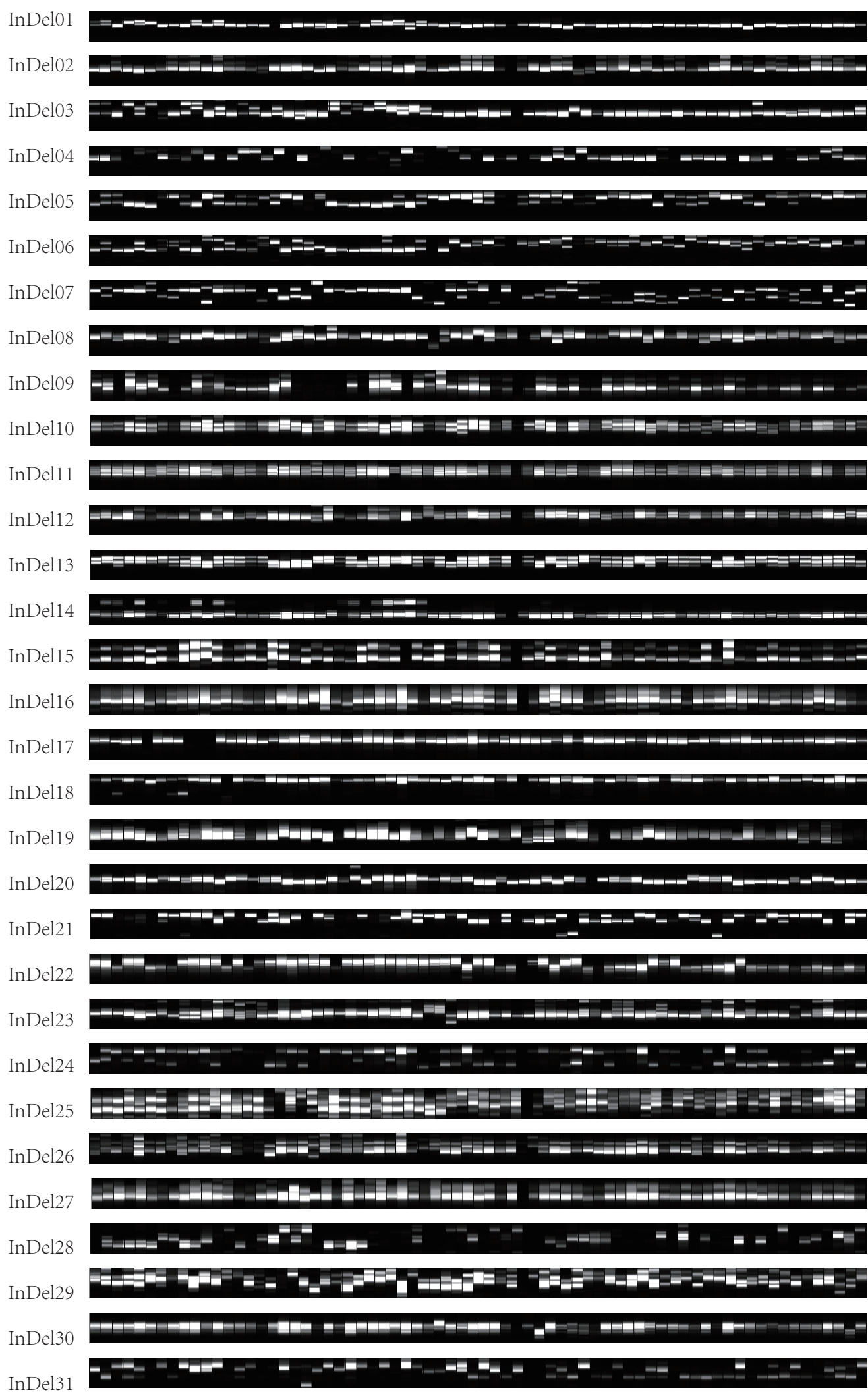

Supplement: Supplementary Figure 4 — Amplification polymorphism of 31 pairs of indel markers among the 69 germplasm samples. [file Data_Sheet_4.PDF]

$$\text{DeltaK} = \text{mean}(|L''(K)|) / \text{sd}(L(K))$$

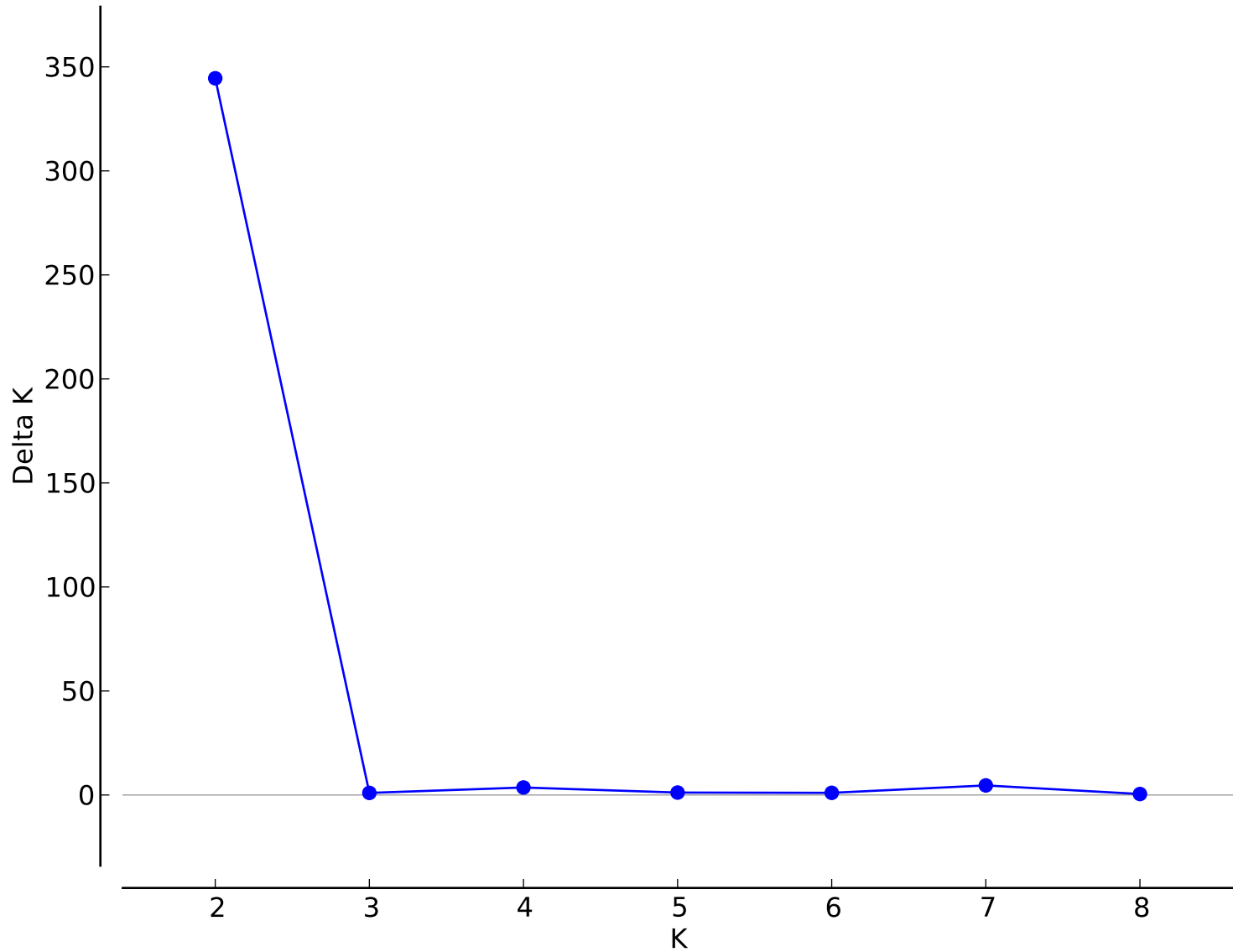

Supplement: Supplementary Figure 5 — Estimation of the optimal group number through ΔK. The value of K was set from 2 to 8. [file Data_Sheet_5.PDF]
