# Supplementary material for: QTL Mapping for Leaf Area of Tea Plants (Camellia sinensis) Based on a High-Quality Genetic Map Constructed by Whole Genome Resequencing
Source: Front Plant Sci. 2021 Jul 29;12:705285. doi: 10.3389/fpls.2021.705285 (PMC8358608; doi:10.3389/fpls.2021.705285)
Supplement: Supplementary Figure 1 — Location information of markers in the genetic map. [file Data_Sheet_1.PDF]

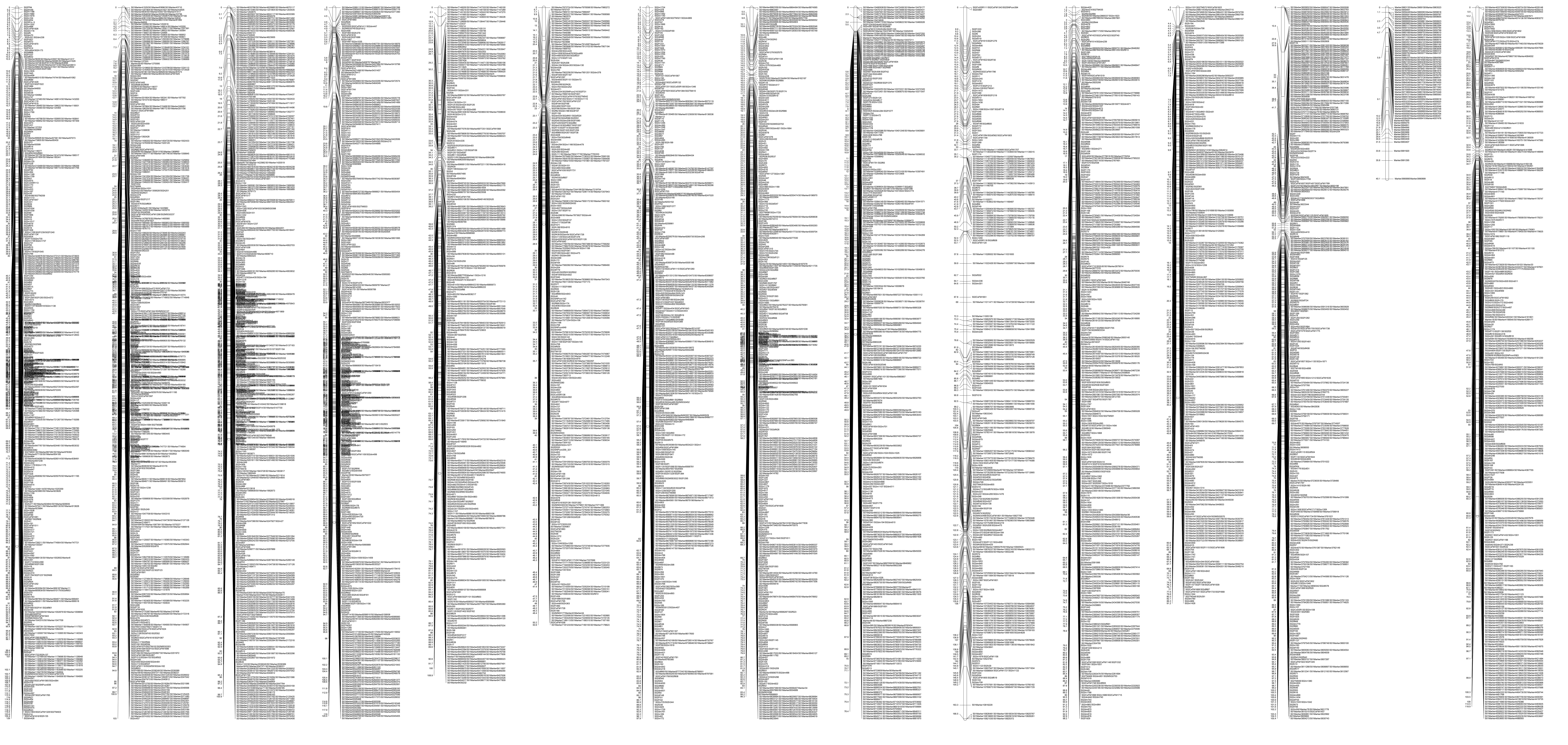
Chr1
Chr2
Chr3
Chr4
Chr5
Chr6
Chr7
Chr8
Chr9
Chr10
Chr11
Chr12
Chr13
Chr14
Chr15
